# Concomitant 5‐Aminosalicylic Acid Does Not Affect the Efficacy of Janus Kinase Inhibitors in Ulcerative Colitis

**DOI:** 10.1002/cpt.70256

**Published:** 2026-03-05

**Authors:** Antonio Tursi, Andrea Pasta, Walter Elisei, Brigida Barberio, Giammarco Mocci, Giovanni Maconi, Antonietta Gerarda Gravina, Raffaele Pellegrino, Giorgia Bodini, Edoardo Vincenzo Savarino, Alfredo Papa, Italian Network for Inflammatory Bowel Diseases (IN‐IBD), Davide G. Ribaldone, Antonio Ferronato, Greta Lorenzon, Davide Checchin, Francesca Lambroglia, Francesco Ferrara, Carla Felice, Giovanni Cataletti, Giorgia Bodini, Andrea Pasta, Giovanni Aragona, Patrizia Perazzo, Federica Gaiani, Stefano Kayali, Fabio Cortellini, Francesco Costa, Lorenzo Bertani, Antonella Scarcelli, Mariaelena Serio, Emanuele Bendia, Laura Bolognini, Daniele Balducci, Claudia Quatraccioni, Francesco Martini, Michele Montori, Simona Piergallini, Francesca Maria Onidi, Paolo Usai Satta, Giorgia Orrù, Raffaele Colucci, Francesco Bachetti, Gabrio Bassotti, Elisabetta Antonelli, Costantino Zampaletta, Giulia Rocco, Carlotta Sacchi, Franco Scaldaferri, Daniele Napolitano, Roberto Faggiani, Michela Di Fonzo, Rita Monterubbianesi, Cristiano Pagnini, Maria Giovanna Graziani, Maria Carla Di Paolo, Roberta Pica, Maddalena Zippi, Andrea Cocco, Claudio Cassieri, Roberto Lorenzetti, Gian Marco Giorgetti, Valeria Clemente, Patrizio Scarozza, Girolamo Bevevino, Giulia Zerboni, Federico Iacopini, Giacomo Forti, Laurino Grossi, Serafina Fiorella, Giovanni Lombardi, Marta Patturelli, Giuliana Vespere, Silvia Sedda, Vittorio D’Onofrio, Leonardo De Luca, Caterina Mucherino, Elvira D’Antonio, Laura Montesano, Pietro Capone, Guido Daniele Villani, Antonio Cuomo, Laura Donnarumma, Nicola Della Valle, Giuseppe Pranzo, Paolo Tonti, Viviana Neve, Libera Fanigliulo, Leonardo Allegretta, Alessia Immacolata Cazzato, Stefano Scorza, Manuela Marzo, Ileana Luppino, Francesco Luzza, Rocco Spagnuolo, Stefano Rodinò, Ladislava Sebkova, Antonio De Medici, Domenico Catarella, Dario D’Agostino, Elisabetta Di Bartolo

**Affiliations:** ^1^ Territorial Gastroenterology Service, Barletta‐Andria‐Trani (BAT) Local Health Agency Andria Italy; ^2^ Department of Translational Medicine and Surgery, School of Medicine Catholic University Rome Italy; ^3^ Division of Gastroenterology Azienda Ospedale‐Università “San Martino”, University of Genoa Genoa Italy; ^4^ Division of Gastroenterology A.O. “S. Camillo‐Forlanini” Hospital Rome Italy; ^5^ Gastroenterology Unit Azienda Ospedale‐Università di Padova (AOUP) Padua Italy; ^6^ Division of Gastroenterology AORN “Brotzu” Hospital Cagliari Italy; ^7^ Gastroenterology Unit, Department of Biomedical and Clinical Sciences “L. Sacco” University Hospital Milan Italy; ^8^ Hepatogastroenterology Division, Department of Precision Medicine University of Campania “Luigi Vanvitelli” Naples Italy; ^9^ Digestive Diseases Centre (CEMAD), Department of Medical and Surgical Sciences, Policlinico Universitario “A. Gemelli” Foundation, IRCCS Rome Italy

## Abstract

We evaluated whether concomitant 5‐aminosalicylic acid (5‐ASA) influences clinical remission in patients with ulcerative colitis (UC) receiving Janus kinase inhibitors (JAKi). In this retrospective, multicenter cohort study, UC patients receiving tofacitinib (*n* = 181), upadacitinib (*n* = 313), or filgotinib (*n* = 139) were included. Time to clinical remission was analyzed using interval‐censored Cox models. Among the 633 patients treated with JAKi, 476 patients received 5‐ASA, and 151 did not. Cumulative probability of remission at week 48 was similar with and without 5‐ASA, at 81.3% and 77.0%, respectively. The adjusted hazard ratio for 5‐ASA vs. no 5‐ASA was 1.12 (95% CI 0.91–1.37, *P* = 0.519). We therefore found that concomitant 5‐ASA did not significantly affect the time to clinical remission in UC patients treated with JAKi.

**Study Highlights**

**WHAT IS THE CURRENT KNOWLEDGE ON THE TOPIC?**

The use of 5‐aminosalicylic acid (5‐ASA) is currently recommended for patients with mild‐to‐moderate ulcerative colitis (UC), but it does not affect therapeutic response in patients treated with concomitant biologic therapy.

**WHAT QUESTION DID THIS STUDY ADDRESS?**

We assessed whether the concomitant use of 5‐ASA may impact the remission rate in UC patients treated with Janus kinase inhibitors (JAKi).

**WHAT DOES THIS STUDY ADD TO OUR KNOWLEDGE?**

The concomitant use of 5‐ASA does not affect the remission rate in UC patients treated with JAKi.

**HOW MIGHT THIS CHANGE CLINICAL PHARMACOLOGY OR TRANSLATIONAL SCIENCE?**

It appears feasible and safe to discontinue 5‐ASA when starting JAKi therapy in patients with UC. However, further studies are needed to investigate whether this concomitant treatment is proper to reduce the risk of colorectal cancer.

In recent years, as drug development and use have advanced, the attention of researchers and physicians managing inflammatory bowel disease (IBD) has also shifted to the usefulness of concomitant background medications. Previous studies have shown that some concomitant drugs (such as corticosteroids, thiopurines, and antibiotics) are associated with biologic agent durability, effectiveness, and immunogenicity risks.^1^, ^2^, ^3^, ^4^ In contrast, concomitant proton pump inhibitor (PPI) use has been associated with lower rates of clinical remission and hospitalization.^4^

Aminosalicylates (5‐ASA) are the most prescribed therapy for mild‐to‐moderate ulcerative colitis (UC),^5^ and often their use also continues after the escalation to advanced drugs. There is evidence that concomitant 5‐ASA treatment does not influence the outcome of UC treated with biologics or immunosuppressant drugs.^6^, ^7^, ^8^, ^9^ Janus kinase inhibitors (JAKi) were found effective in managing UC.^10^ However, there is a lack of information about the impact of concomitant 5‐ASA therapy in patients taking JAKi for the management of UC. In addition, no evidence of clinically relevant pharmacodynamic or pharmacokinetic interaction between 5‐ASA and JAKi was found. Hence, we conducted a real‐world analysis of a large population treated with JAKis and 5‐ASA for UC.

## PATIENTS AND METHODS

We retrospectively analyzed anonymized records of UC patients treated with JAKi (tofacitinib, upadacitinib, filgotinib) from multiple Italian centers. Concomitant 5‐ASA use was recorded at baseline and weeks 8, 24, and 48. The minimal 5‐ASA oral dosage considered in the study was 1,600 mg/day. Demographics (e.g., birth‐assigned sex, age, smoking status), disease history (comorbidities, age at UC diagnosis, previous treatments undertaken), and clinical variables such as the partial Mayo score (PMS),^11^ fecal calprotectin, and C‐reactive protein (CRP) were collected. Clinical remission was defined as a partial Mayo score ≤1.^11^

This study adhered to the Declaration of Helsinki (1975) and the Italian Medicines Agency's determination of 20 March 2008. Ethical approval was not required due to its retrospective, observational, and anonymous design. All data were strictly anonymized, analyzed in aggregate form, and could not be traced back to individual patients. The manuscript was prepared following the guidelines for strengthening the reporting of observational studies in epidemiology (STROBE).^12^

Baseline characteristics were compared using chi‐square, ANOVA, or Kruskal–Wallis tests as appropriate. Stabilized inverse probability of treatment weighting (IPTW) from a multinomial propensity score incorporating demographics, severity, prior therapies, and covariates achieved balance (standardized mean <0.10). Interval‐censored proportional hazards estimated time to remission. Drug‐stratified models used an α of 0.05, reporting hazard ratios and 95% confidence intervals.

## RESULTS

Overall, the cohort included 633 patients treated with filgotinib (FILGO, *n* = 139), tofacitinib (TOFA, *n* = 181), or upadacitinib (UPA, *n* = 313). Before IPTW, groups differed for age (47.9 ± 15.4; 46.1 ± 14.1; 43.1 ± 13.7 years, respectively; *P* = 0.002) and prior advanced therapies (JAK‐i 9.4%, 8.3%, 16.6%; *P* = 0.011; anti‐TNF 89.2%, 89.0%, 95.2%; *P* = 0.017; vedolizumab 35.3%, 53.5%, 39.0%; *P* = 0.001; ustekinumab 25.2%, 11.6%, 20.4%; *P* = 0.007). Table I reports the clinical and demographic characteristics of the enrolled population, which was virtually free of comorbidities. The prevalence of 5‐ASA use at baseline was similar across groups (FILGO: 74.1%; TOFA: 71.3%; UPA: 75.4%; *P* = 0.601). After IPTW, the weighted pseudopopulation comprised 627 patient equivalents (FILGO *n* = 138; TOFA *n* = 179; UPA *n* = 310) (**Table** **1**). Concomitant 5‐ASA use was widespread and remained similar across treatment arms, with FILGO (*n* = 108/138; 78.0%), TOFA (*n* = 129/179; 72.3%), and UPA (*n* = 240/310; 77.1%) showing no between‐group differences (*P* = 0.495).

**Table 1:** Baseline characteristics of study population by treatment group after IPTW

	FILGO, *n* = 138	TOFA, *n* = 179	UPA, *n* = 310	*P*‐value
Sex, male	68 (49.1)	103 (57.5)	189 (61)	0.124
Age at study inclusion, years	45 ± 15.2	46 ± 14.6	44.9 ± 13.9	0.607
Disease duration, years	9.8 ± 8.2	10.4 ± 7.2	10.5 ± 8.5	0.997
Active smoker	13 (9.5)	14 (7.8)	43 (14.2)	0.075
Previous surgery	5 (3.7)	3 (1.7)	8 (2.6)	0.621
Charlson comorbidity index, points	0 ± 0	0 ± 0	0 ± 1	0.985
Extensive colitis	89 (64.8)	116 (64.9)	186 (59.9)	0.811
Previous biologic therapies				
Jak‐i	17 (12.2)	20 (11.2)	43 (14)	0.728
TNF‐i	124 (90.2)	164 (91.6)	288 (92.6)	0.756
Vedolizumab	56 (40.5)	79 (44)	126 (40.4)	0.776
Ustekinumab	27 (19.7)	27 (15.1)	61 (19.7)	0.506
Therapy at inclusion				
Steroids	53 (38.4)	75 (42)	132 (42.3)	0.777
5‐ASA	108 (78)	129 (72.3)	240 (77.1)	0.495
Thiopurine	1 (0.7)	3 (1.9)	14 (4.4)	0.073
Disease activity at inclusion				
Mayo score, points	6 ± 2	6 ± 3	6 ± 2	0.808
Fecal calprotectin, μg/g	1031.7 ± 1539.3	1121.7 ± 1303.4	963.6 ± 1126.8	0.402
C‐reactive protein, mg/dL	10.7 ± 10.3	9.5 ± 13.9	11 ± 13.6	0.098
Endoscopic Mayo score, points	2 ± 1	2 ± 1	2 ± 1	0.423

Data are shown as absolute values and frequencies, and as the median and interquartile range.

**Figure 1:**
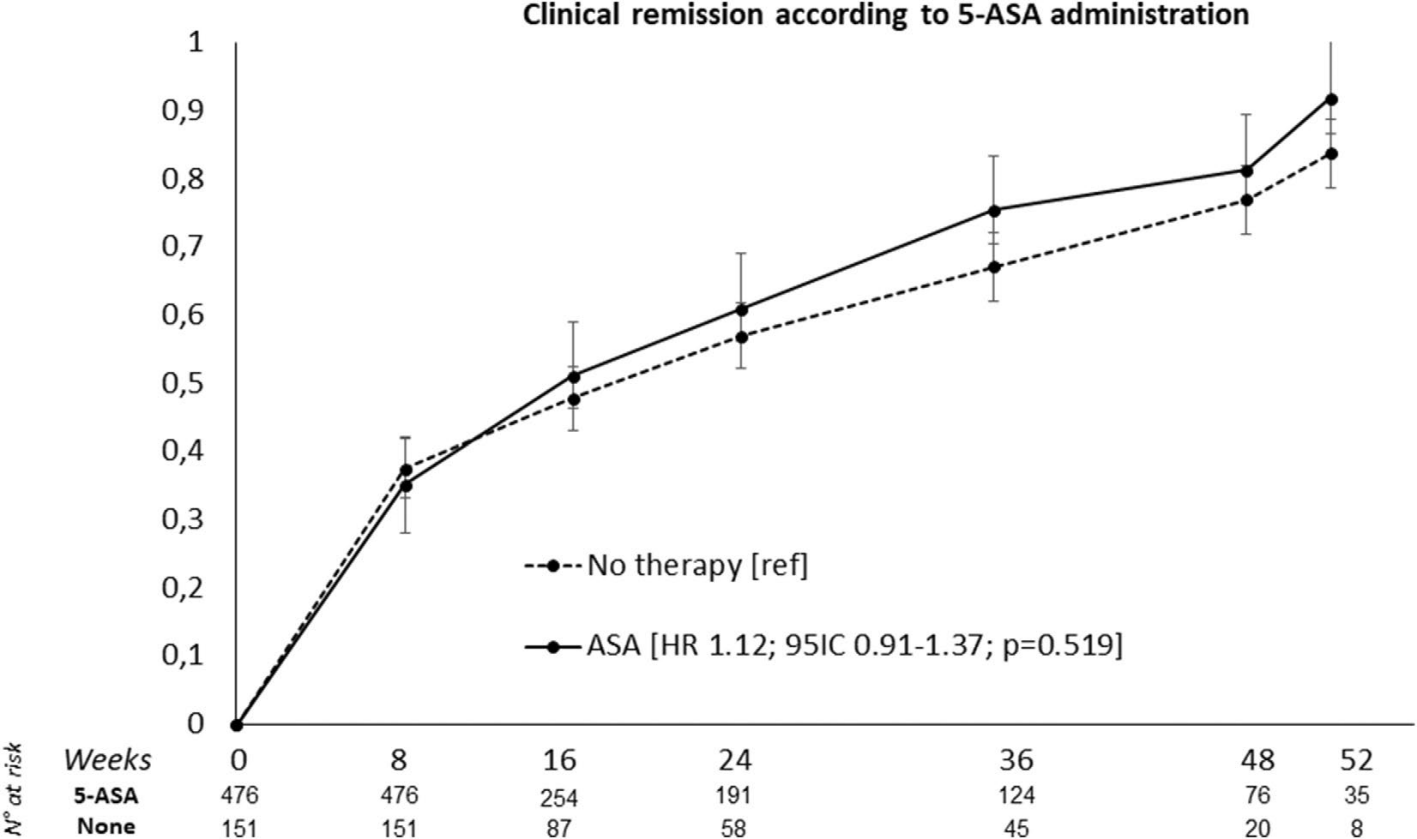
Clinical remission according to 5‐ASA concomitant treatment in UC patients treated with JAKi.

In the interval‐censored Cox analysis (**Figure** **1**), the cumulative probability of achieving clinical remission in patients receiving 5‐ASA vs. those not receiving it was 35.2% (95% CI 28.1–42.3, *n*‐at‐risk 476) vs. 37.5% (33.2–41.9, *n*‐at‐risk 151) at week 8, 51.1% (43.3–55.8, *n*‐at‐risk 254) vs. 47.9% (43.2–52.6, *n*‐at‐risk 87) at week 16, 75.4% (67.4–80.4, *n*‐at‐risk 124) vs. 67.1% (62.2–72.1, *n*‐at‐risk 45) at week 36, and 81.3% (73.0–86.2, *n*‐at‐risk 76) vs. 77.0% (72.0–81.9, *n*‐at‐risk 20) at week 48, and the corresponding hazard ratio for 5‐ASA vs. no 5‐ASA was 1.12 (95% CI 0.91–1.37, *P* = 0.519), indicating no statistically significant difference in time to remission.

In drug‐stratified interval‐censored Cox models comparing patients receiving 5‐ASA with those not receiving 5‐ASA, the hazard ratio for time to clinical remission was 0.98 (95% CI 0.60–1.60, *P* = 0.922) for TOFA, 1.25 (0.98–1.59, *P* = 0.068) for UPA, and 1.14 (0.76–1.73, *P* = 0.519) for FILGO, with no statistically significant differences within individual cohorts.

Safety was comparable between patients receiving 5‐ASA and those not receiving 5‐ASA, with similar rates of any AE (140, 29.4% vs 43, 28.7%; *P* = 0.873); no statistically significant differences were observed for infections of any type (62, 13.0% vs 21, 14.0%; *P* = 0.752), upper respiratory tract infections (40, 8.4% vs 14, 9.3%; *P* = 0.718), herpes zoster (14, 2.9% vs 5, 3.3%; *P* = 0.804), transaminase elevations (13, 2.7% vs 5, 3.3%; *P* = 0.697), or serious adverse events (18, 3.8% vs 6, 4.0%; *P* = 0.900).

## DISCUSSION

While 5‐ASA remains the cornerstone of treatment for mild‐to‐moderate UC,^12^ its utility as an adjunctive therapy with JAKi or biologics is increasingly debated. Several recent studies found no effect of continued 5‐ASA on maintaining remission with biologics.^6^, ^7^, ^8^, ^9^ Our study is the first to analyze the impact of 5‐ASA during JAKi therapy using real‐world data and shows no additional benefit. This result is not fully surprising. If persistent inflammation is the major risk factor for treatment failure and advanced drugs effectively control inflammation,^13^ the addition of 5‐ASA becomes clinically negligible for remission induction or maintenance.

A significant limitation of our analysis was that we were unable to assess whether topical 5‐ASA contributed to the outcome (data were not recorded). However, we believe this is unlikely, as topical 5‐ASA is typically prescribed for a short term due to limited compliance with such treatment.

A key issue regarding the concomitant use of 5‐ASA in UC patients receiving JAKi is the potential to exploit its chemopreventive effect, although this role remains debated.^14^ Because persistent intestinal inflammation is a significant risk factor for the development of colorectal cancer in IBD, any therapy capable of inducing and maintaining mucosal healing—such as biologics and small molecules—will likely also decrease the risk of IBD‐associated colorectal cancer.^15^ However, until this is clearly demonstrated, it seems appropriate that the indications for the concomitant use of 5‐ASA remain at least for the more severe and extensive forms of the disease, forms with a higher risk of developing CRC.^16^

In conclusion, we found that concomitant 5‐ASA use after initiating JAKi in UC does not improve clinical outcomes. It appears feasible and safe to discontinue 5‐ASA when escalating to JAKi. Furthermore, prospective studies investigating the short‐ and long‐term benefits and risks of 5‐ASA discontinuation after JAKi initiation are needed to confirm our findings.

## AUTHOR CONTRIBUTIONS

A.T. wrote the manuscript. A.T., W.E., B.B., G.Mo., and A.Papa designed the research. A.T., A.Pasta, W.E., and A.Papa performed the research; A.Pasta analyzed the data; A.T., A.Pasta, W.E., B.B., G.Mo., G.Ma., A.G.G., R.P., G.B., E.V.S., and A.Papa contributed new reagents/analytical tools; A.T.: Antonio Tursi; A. Pasta; Andrea Pasta; W.E.: Walter Elisei, B.B.: Brigida Barberio; Gi.Mo.: Giammarco Mocci; Gi.Ma.: Giovanni Maconi; A.G.G.: Antonietta Gerarda Gravina; R.P.: Raffaele Pellegrino; G.B.: Giorgia Bodini; E.V.S.: Edoardo Vincenzo Savarino; A. Papa: Alfredo Papa. All authors approved the version to be published.

## FUNDING

No funding was received for this work.

## CONFLICT OF INTEREST

Antonio Tursi has served as a speaker and/or consultant for AbbVie, Bayer, Fenix Pharma, Galápagos, Janssen, Nalkein, Omega Pharma, and SILA; Giammarco Mocci has served as speaker for and/or received advisory board fees from AbbVie, Amgen, Aurora Biopharma, Biogen, Celltrion, Chiesi, Fenix Pharma, Ferring, Galápagos, Janssen, MSD, Omega Pharma, Sandoz, Takeda, and Vifor Pharma; Edoardo V. Savarino has served as a speaker for AbbVie, AGPharma, Alfasigma, Dr Falk, EG Stada Group, Fresenius Kabi, Grifols, Janssen, Innovamedica, Malesci, Pfizer, Reckitt Benckiser, Sandoz, SILA, Sofar, Takeda, and Unifarco and has served as consultant for Alfasigma, Amgen, Biogen, Bristol‐Myers Squibb, Celltrion, Diadema Farmaceutici, Dr Falk, Fresenius Kabi, Janssen, Merck & Co., Reckitt Benckiser, Regeneron, Sanofi, Shire, SILA, Sofar, Synformulas GmbH, Takeda, and Unifarco; he received research support from Pfizer, Reckitt Benckiser, SILA, Sofar, and Unifarco; Giovanni Maconi has served as a speaker for and/or has received advisory board fees from AlfaSigma, Arena, Janssen, Gilead, and Roche; Alfredo Papa received speaker fees from Alfasigma and Janssen; Antonietta Gerarda Gravina has conducted training activities (e.g., ECM, preceptorship) for Pfizer, Galápagos Biopharma, and AbbVie; Raffaele Pellegrino has received sponsorship for participation in national and/or international conferences from Pfizer Inc., Eli Lilly, Alfasigma, and Abbvie; Andrea Pasta, Walter Elisei, Brigida Barberio, and Giorgia Bodini have no conflicts of interest to declare.

## Appendix A

Davide G. Ribaldone: IBD Unit, A.O.U. Citta della Salute e della Scienza”, Torino; Antonio Ferronato: UOSD di Endoscopia Digestiva, ULSS7 Pedemontana, Ospedale di Santorso, Santorso (VI); Greta Lorenzon: UOC di Gastroenterologia, Dipartimento di Scienze Chirurgiche, Oncologiche e Gastroenterologiche, Università di Padova, Azienda Ospedaliero‐Universitaria “Sant'Antonio”, Padova; Davide Checchin, Francesca Lambroglia, Francesco Ferrara: UOC di Gastroenterologia, Ospedale” S Giovanni e Paolo”, Mestre – Venezia; Carla Felice: UOC di Medicina Interna, Ospedale Universitario “Ca′ Foncello”, Treviso; Giovanni Cataletti: UOC di Gastroenterologia, A.O. “L. Sacco”, Milano; Giorgia Bodini, Andrea Pasta: UOC di Gastroenterologia, Dipartimento di Medicina Interna, IRCCS Ospedale “San Martino” Hospital, Università di Genova, Genova; Giovanni Aragona, Patrizia Perazzo: UOC di Gastroenterologia, Ospedale “Guglielmo da Saliceto”, Piacenza; Federica Gaiani, Stefano Kayali: UOC di Gastroenterologia ed Endoscopia Digestiva, Dipartimento di Medicina e Chirurgia, Ospedale Maggiore, Università di Parma, Parma; Fabio Cortellini: UOC di Gastroenterologia ed Endoscopia Digestiva, Ospedale” Infermi", Rimini; Francesco Costa: UOC di Gastroenterologia, Azienda Ospedaliero‐Universitaria Pisana, Pisa; Lorenzo Bertani: UOC di Gastroenterologia, Ospedale “Felice Lotti”, Azienda USL Toscana Nord Ovest, Pontedera (PI); Antonella Scarcelli, Mariaelena Serio: UOC di Gastroenterologia, Ospedale “San Salvatore”, Pesaro; Emanuele Bendia, Laura Bolognini, Daniele Balducci, Claudia Quatraccioni, Francesco Martini, Michele Montori: UOC di Gastroenterologia, Endoscopia Digestiva e Malattia infiammatorie Croniche Intestinali, Ospedale “Torrette”, Ancona; Simona Piergallini: UOC di Gastroenterologia, IBD Unit, Ospedale “A. Murri”, Fermo; Francesca Maria Onidi, Paolo Usai Satta: UOC di Gastroenterologia, Azienda Ospedaliera di Rilievo Nazionale “Brotzu”, Cagliari; Giorgia Orrù: University of Cagliari, Department of Medical Sciences and Public Health, Cagliari, Italy; Raffaele Colucci, Francesco Bachetti: UOSD di Endoscopia Digestiva, Ospedale “San Matteo degli Infermi", Spoleto (PG); Gabrio Bassotti, Elisabetta Antonelli: Sezione di Gastroenterologia ed Epatologia, Dipartimento di Medicina, Ospedale “Santa Maria della Misericordia”, Perugia; Costantino Zampaletta, Giulia Rocco, Carlotta Sacchi: UOC di Gastroenterologia, Ospedale “Belcolle”, Viterbo; Franco Scaldaferri, Daniele Napolitano: UOC di Medicina Interna e Gastroenterologia, Dipartimento di Scienze Mediche e Chirurgiche, IRCCS Fondazione “Policlinico Gemelli, Università Cattolica del Sacro Cuore, Roma; Roberto Faggiani, Michela Di Fonzo, Rita Monterubbianesi: UOC di Gastroenterologia ed Endoscopia Digestiva, A.O. “S. Camillo‐Forlanini”, Roma; Cristiano Pagnini, Maria Giovanna Graziani, Maria Carla Di Paolo: UOC di Gastroenterologia ed Endoscopia Digestiva, A.O. “S. Giovanni – Addolorata”, Roma; Roberta Pica, Maddalena Zippi, Andrea Cocco, Claudio Cassieri: UOC di Gastroenterologia ed Endoscopia Digestiva, Ospedale “S. Pertini”, Roma; Roberto Lorenzetti: UOC di Gastroenterologia, Ospedale Territoriale “Nuovo Regina Margherita”, Roma; Gian Marco Giorgetti, Valeria Clemente: UOSD di Endoscopia Digestiva Nutrizionale, Ospedale “S. Eugenio”, Roma; Patrizio Scarozza, Girolamo Bevevino, Giulia Zerboni, Federico Iacopini: UOC di Gastroenterologia ed Endoscopia Digestiva, “Ospedale dei Castelli”, Ariccia (Roma); Giacomo Forti: UOSD di Endoscopia Digestiva, Ospedale “S. Maria Goretti”, Latina; Laurino Grossi: UOC di Gastroenterologia, Ospedale “Spirito Santo”, Università “G d'Annunzio”, Pescara; Serafina Fiorella: UOSD di Gastroenterologia ed Endoscopia Digestiva, Ospedale “P. Pio”, Vasto (CH); Giovanni Lombardi, Marta Patturelli: UOC di Gastroenterologia, Azienda Ospedaliera di Rilievo Nazionale “Antonio Cardarelli”, Napoli; Giuliana Vespere, Silvia Sedda, Vittorio D'Onofrio, Leonardo De Luca: UOC di Gastroenterologia, “Ospedale del Mare” ASL NA1 Centro, Napoli; Caterina Mucherino, Elvira D'Antonio, Laura Montesano: UOC di Gastroenterologia, Azienda Ospedaliera “S. Anna e S. Sebastiano”, Caserta; Pietro Capone, Guido Daniele Villani: UOC di Gastroenterologia ed Endoscopia Digestiva, Ospedale “T. Maresca”, Torre del Greco (NA); Antonio Cuomo, Laura Donnarumma: UOSD di Gastroenterologia, Ospedale “Umberto I”, Nocera Inferiore (SA); Nicola Della Valle: UOC di Gastroenterologia, A.O. “Ospedali Riuniti”, Foggia; Giuseppe Pranzo: UOS di Endoscopia Digestiva, Ospedale “Valle D'Itria”, Martina Franca (TA); Paolo Tonti, Viviana Neve: UOSD di Gastroenterologia ed Endoscopia Digestiva, Ospedale “A. Perrino”, Brindisi; Libera Fanigliulo: UOC di Gastroenterologia, A.O. “S.S. Annunziata”, Taranto; Leonardo Allegretta, Alessia Immacolata Cazzato, Stefano Scorza: UOC di Gastroenterologia ed Endoscopia Digestiva, Ospedale “Santa Caterina Novella”, Galatina (LE); Manuela Marzo: UOC di Gastroenterologia ed Endoscopia Digestiva, Ospedale “Veris‐Delli Ponti”, Scorrano (LE); Ileana Luppino: UOC di Gastroenterologia ed Endoscopia Digestiva, Ospedale “Annunziata”, Cosenza; Francesco Luzza, Rocco Spagnuolo: Dipartimento di Scienze Mediche, UOC di Fisiopatologia Digestiva, Azienda Ospedaliera “Mater Domini”, Università di Catanzaro, Catanzaro; Stefano Rodinò, Ladislava Sebkova: UOC di Gastroenterologia, A.O. “Ciaccio‐Pugliese”, Catanzaro; Antonio De Medici: Servizio di Gastroenterologia Territoriale, AST Catanzaro, Catanzaro Lido; Domenico Catarella, Dario D'Agostino, Elisabetta Di Bartolo: UOC di Gastroenterologia, ARNAS “Garibaldi”, Catania.

## References

[1] Faleck, D.M. , Shmidt, E. , Huang, R. *et al*. Effect of concomitant therapy with steroids and tumor necrosis factor antagonists for induction of remission in patients with Crohn's disease: a systematic review and pooled meta‐analysis. Clin. Gastroenterol. Hepatol. 19, 238–245 (2021). 32569749 10.1016/j.cgh.2020.06.036 PMC8364422

[2] Gorelik, Y. , Freilich, S. , Gerassy‐Vainberg, S. *et al*. Antibiotic use differentially affects the risk of anti‐drug antibody formation during anti‐TNFalpha therapy in inflammatory bowel disease patients: a report from the epi‐IIRN. Gut 71, 287–295 (2022). 34344783 10.1136/gutjnl-2021-325185 PMC8762017

[3] Gorelik, Y. *et al*. Association of Antibiotic use with durability of biologic agents in inflammatory bowel disease: a report from the epi‐IIRN. J. Crohns Colitis 17, 1410–1417 (2023). 37084088 10.1093/ecco-jcc/jjad070

[4] Lu, T.X. , Dapas, M. , Lin, E. , Peters, T. & Sakuraba, A. The influence of proton pump inhibitor therapy on the outcome of infliximab therapy in inflammatory bowel disease: a patient‐level meta‐analysis of randomised controlled studies. Gut 70, 2076–2084 (2021). 33334900 10.1136/gutjnl-2020-321609

[5] Raine, T. *et al*. ECCO guidelines on therapeutics in ulcerative colitis: medical treatment. J. Crohns Colitis 16, 2–17 (2022). 34635919 10.1093/ecco-jcc/jjab178

[6] Ungaro, R.C. *et al*. Stopping 5‐aminosalicylates in patients with ulcerative colitis starting biologic therapy does not increase the risk of adverse clinical outcomes: analysis of two nationwide population‐based cohorts. Gut 68, 977–984 (2019). 30420398 10.1136/gutjnl-2018-317021 PMC7057119

[7] Choi, Y.I. *et al*. Comparison of outcomes of continuation/discontinuation of 5‐aminosalicylic acid after initiation of anti‐tumor necrosis factor‐alpha therapy in patients with inflammatory bowel disease. Int. J. Color. Dis. 34, 1713–1721 (2019). 10.1007/s00384-019-03368-1 31471699

[8] Bernstein, C.N. , Tennakoon, A. , Singh, H. & Targownik, L. Continued 5ASA use after initiation of anti‐TNF or immunomodulator confers no benefit in IBD: a population‐based study. Aliment. Pharmacol. Ther. 54, 814–832 (2021). 34247410 10.1111/apt.16518

[9] Ahuja, D. , Zou, G. , Solitano, V. *et al*. S.No impact of concomitant medications on efficacy and safety of biologics and small molecules for ulcerative colitis. Clin. Gastroenterol. Hepatol. 23, 1786–1797 (2025). 39395572 10.1016/j.cgh.2024.08.040 PMC12186698

[10] Konzett, V. *et al*. Efficacy of Janus kinase inhibitors in immune‐mediated inflammatory diseases: a systematic literature review informing the 2024 update of an international consensus statement. Ann. Rheum. Dis. 84, 680–696 (2025). 39934019 10.1016/j.ard.2025.01.023

[11] Lewis, J.D. , Chuai, S. , Nessel, L. , Lichtenstein, G.R. , Aberra, F.N. & Ellenberg, J.H. Use of the noninvasive components of the Mayo score to assess clinical response in ulcerative colitis. Inflamm. Bowel Dis. 14, 1660–1666 (2008). 18623174 10.1002/ibd.20520 PMC2597552

[12] Cuschieri, S. The STROBE guidelines. Saudi J. Anaesth. 13, S31–S34 (2019). 30930717 10.4103/sja.SJA_543_18 PMC6398292

[13] Cai, X.X. *et al*. A comprehensive review of small molecules, targets, and pathways in ulcerative colitis treatment. Eur. J. Med. Chem. 291, 117645 (2025). 40279769 10.1016/j.ejmech.2025.117645

[14] Herfarth, H. The role of chemoprevention of colorectal cancer with 5‐aminosalicylates in ulcerative colitis. Dig. Dis. 30(Suppl 2), 55–59 (2012). 10.1159/000341894 23207933

[15] Herfarth, H. & Vavricka, S.R. 5‐Aminosalicylic acid chemoprevention in inflammatory bowel diseases: is it necessary in the age of biologics and small molecules? Inflamm. Intest. Dis. 7, 28–35 (2021). 35224015 10.1159/000518865 PMC8820128

[16] Shah, S.C. & Itzkowitz, S.H. Colorectal cancer in inflammatory bowel disease: mechanisms and management. Gastroenterology 162, 715–730 (2022). 34757143 10.1053/j.gastro.2021.10.035 PMC9003896

